# Assessing the blood meal hosts of *Culex quinquefasciatus* and *Aedes taeniorhynchus* in Isla Santa Cruz, Galápagos

**DOI:** 10.1186/s13071-019-3835-7

**Published:** 2019-12-16

**Authors:** Samoa Asigau, Sawsan Salah, Patricia G. Parker

**Affiliations:** 1 Department of Biology, University of Missouri, St. Louis, One University Blvd., St. Louis, MO 63121 USA; 2 Whitney R. Harris World Ecology Center, University of Missouri, One University Blvd., St. Louis, MO 63121 USA; 3 WildCare Institute, Saint Louis Zoo, One Government Drive, St. Louis, MO 63110 USA

**Keywords:** Mosquito, Feeding patterns, Galápagos, *Aedes*, *Culex*, Santa Cruz

## Abstract

**Background:**

Blood meal host selection by mosquito vectors is an important component in understanding disease dynamics of pathogens that threaten endemic fauna in isolated islands such as Galápagos. Research on the feeding behavior of mosquitoes can provide clues to the hosts and vectors involved in disease transmission. This information is particularly critical for endemic wildlife fauna in island systems that have evolved without resistance to novel diseases such as avian malaria. The aims of this study were to determine the blood-feeding patterns of two species of mosquitoes found in Galápagos and discuss how their feeding behavior may influence the transmission of pathogens such as avian malaria.

**Methods:**

In the summer of 2015, we sampled two mosquito species (*Aedes taeniorhynchus* and *Culex quinquefasciatus*) across 18 different sites on Isla Santa Cruz, which is the second largest island in Galápagos and has the largest human population. We trapped mosquitoes using CDC light traps and CDC gravid traps and identified sources of blood meals for engorged mosquitoes by sequencing a portion of the vertebrate mitochondrial cytochrome *b* gene.

**Results:**

Out of 947 female mosquitoes captured, 320 were blood-fed, and PCR amplifications were successful for 301 of the blood meals. Results revealed that both *Aedes taeniorhynchus* and *Culex quinquefasciatus* feed from a variety of vertebrate taxa, numerically dominated by humans on Isla Santa Cruz.

**Conclusions:**

The high proportion of mammalian blood meals could represent locally available and abundant hosts on Santa Cruz. However, host surveys and estimates of relative abundances of vertebrate species will need to accompany mosquito trapping studies on non-inhabited and inhabited islands in Galápagos to further validate this.

## Background

Knowledge of blood-feeding patterns by mosquitoes can provide an insight into disease dynamics and help manage parasites that pose threats to endemic wildlife. Many insects such as mosquitoes require a blood meal to complete their gonotrophic cycle and can thereby transmit blood-borne pathogens that threaten the health of wildlife and humans [1–3]. Host preference by mosquitoes appears to be heritable [4, 5] but can also depend on ecological factors like host availability, host abundance, vector abundance, habitat and climate [6, 7]. In addition, when hosts become rare or limited, disease vectors may disperse to new habitats and modify their feeding behavior to a more diverse range of hosts. This shift in feeding behavior by disease vectors may have serious implications for disease transmission and dynamics, especially in novel habitats. For instance, numerous endemic birds in Hawaii faced extinction from the co-introduction of avian malaria and avian pox, two virulent pathogens common to birds in continental areas. These parasites were likely carried to Hawaii from continents through migratory birds [8]. The mosquito vector *Culex quinquefasciatus* (Say, 1823) assisted in transmitting deadly pathogens from resistant migrants to naïve native birds, resulting in extinctions of many endemic Hawaiian bird species [1, 9].

The Galápagos Archipelago, located almost 1000 km from the west coast of mainland Ecuador, is similar to Hawaii in terms of its island ecosystem that is volcanic in origin. The islands are known for their high endemism that inspired Charles Darwin’s theory of evolution by natural selection [10]. Given its iconic natural system, the archipelago’s flora and fauna are well studied and human movements and impacts in the archipelago are at least partly controlled and monitored by the collective efforts of the Galápagos National Park and the Charles Darwin Research Station. Despite these efforts, the archipelago already hosts three mosquito vectors, *Cx. quinquefasciatus*, *Aedes aegypti* (L.) and *Aedes taeniorhynchus* (Wiedemann, 1821). Estimated to have naturally arrived ~ 200,000 years ago [11], *Ae. taeniorhynchus* (or the black salt marsh mosquito) oviposits in brackish water [12]. In contrast, *Ae. aegypti* and *Cx. quinquefasciatus* require freshwater for oviposition and have been estimated to have established populations in the archipelago in 2001 and 1985, respectively [13, 14]. *Aedes aegypti* is highly anthropophilic and has been found in human-inhabited zones such as those on Santa Cruz and Isabela [13, 15].

The black salt marsh mosquito, *Ae. taeniorhynchus*, has been shown to have a strong preference for taking blood meals from reptiles and mammals over birds in mosquitoes sampled on uninhabited islands in the Galápagos archipelago [12]. However, it is unknown how its feeding preferences may change on human-inhabited islands. In addition, the blood meal host identities and possible preferences in Galápagos of a recent arrival, *Cx. quinquefasciatus*, remain unknown. Our knowledge of host-parasite associations in Galápagos also remains fragmentary; therefore, studies of feeding behavior by mosquitoes may provide clues to the arthropod vectors involved in disease transmission.

The pathogens transmitted by mosquitoes include the haemosporidian blood parasites in the genus *Plasmodium* that cause avian malaria. Extensive sampling and molecular screening of endemic Galápagos penguin populations (*Spheniscus mendiculus*) revealed *via* PCR the presence of an avian parasite within the genus *Plasmodium* (lineage A) with infections detected in 3–9.4% of sampled penguins per year [16, 17]. However, the absence of gametocytes (stage of the parasite infective to arthropod vectors) within thin blood films prepared from infected penguins suggests parasitic abortive development, indicating that penguins could be dead-end hosts. Three additional *Plasmodium* lineages (B, C, D) have since been discovered along with microscopic detection of a *Plasmodium* erythrocytic meront from a cactus finch (*Geospiza scandens*) and haemosporidian trophozoites from a vegetarian finch (*Platyspiza crassirostris*) [16]. Other arthropod-vectored pathogens known to infect Galápagos birds include several lineages of *Haemoproteus* (Order Haemosporida) [18–21], microfilarid nematodes [22] and avian poxvirus [23].

The transmission of pathogens in Galápagos may involve arthropod vectors such as mosquitoes. Therefore, it is important to understand the blood meal hosts of mosquitoes, which we aim to investigate for two mosquitoes common in Galápagos, *Ae. taeniorhynchus* and *Cx. quinquefasciatus*, and discuss their role in transmitting important pathogens that threaten endemic wildlife in Galápagos.

## Methods

### Study site

This study was conducted on Santa Cruz Island, which is part of the Galápagos archipelago. Consisting of 13 major islands and 19 smaller islands, the archipelago is volcanic in origin and predominantly arid. The islands are known for their high endemism and low biodiversity with 530 species of fish and 111 other vertebrate species of mammals, birds and reptiles. There are 48 species of seabirds of which 19 are resident in Galápagos. Land birds constitute 29 resident species of which 22 are endemic and 4 are endemic to the level of subspecies. There are 25 mammal species consisting of two endemic species and 28 species of reptiles of which 19 are endemic [24].

Our study was conducted on Isla Santa Cruz between May 20th and August 3rd, 2015. Santa Cruz is the second largest island in Galápagos with a land area of 986 km^2^ and is one of four inhabited islands along with Isabela, Floreana and San Cristobal. The 2010 census recorded 15,000 inhabitants on Santa Cruz, making it the largest human population among the islands. This total represents 60% of the archipelago’s human population [25] and nearly double the population of the whole archipelago since 1998. Likewise, the tourism industry has dramatically increased in the late 20th century, especially among inhabited islands. In 1969, approximately 2000 people visited the Galápagos Islands, which is a small fraction of the 180,000 people who visited in 2012 [26]. Compared to other islands, Santa Cruz hosts most of this human population and attracts tourists due to its developed infrastructure such as a hospital, schools, banks, shops, hotels and restaurants. Included in this infrastructure is a single 40 km paved road that extends from the north at Itabaca Channel, which is the entrance to Santa Cruz from the airstrip on adjacent Baltra Island, to the most southern tip at Puerto Ayora. Humans mainly inhabit the southern windward half of Santa Cruz since it provides ideal conditions for agriculture, and towns include Puerto Ayora, Miramar, Bellavista, Santa Rosa and Santa Martha.

### Mosquito survey

**Fig. 1:**
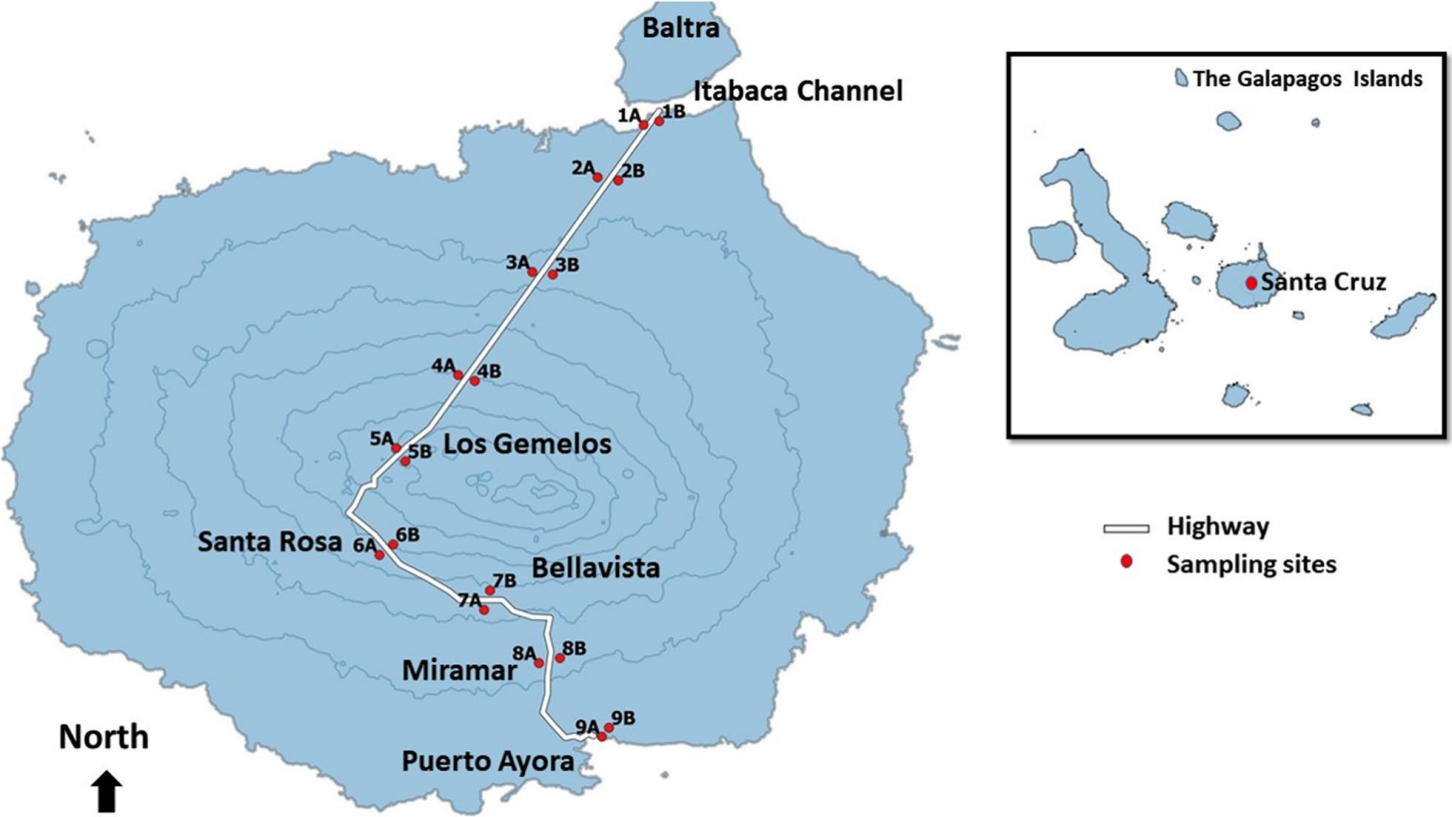
Map of 18 mosquito sampling sites extending from the most northern site, Itabaca Channel to the most southern site, Puerto Ayora. Names of localities (Itabaca Channel, Los Gemelos, Santa Rosa, Bellavista, Miramar and Puerto Ayora) are also indicated beside their corresponding mosquito sampling sites

We trapped mosquitoes across 18 sites along the main highway that stretches from the north at Itabaca Channel to the south at Puerto Ayora. Using the highway as a transect, we established 9 trapping stations spaced 5 km apart and set two trapping locations spaced at 300 m at each station to avoid edge effects, thus totaling 18 independent trapping sites (Fig. 1). At each site, we established a total of 4 points measuring 50 m apart and alternated 2 CDC light traps (Model 512 John Hock Company, Gainesville, USA) and 2 CDC gravid traps (Model 1712 John Hock Company) across these points. CDC light traps were baited with a CO_2_ emitting mixture consisting of 250 g sugar, 35 g yeast and 2.5 liters of water to attract host-seeking mosquitoes [27, 28] and gravid traps were baited with a hay-yeast-water infusion to attract ovipositing mosquitoes [29]. Traps were set within one hour of dusk and mosquitoes were collected in the morning the next day. Mosquitoes were immobilized with chloroform, sexed and identified to species level using morphological characters [30]. We classified female mosquitoes according to the Sella scale (1, unfed; 2–6, partial to full blood meal; 7, gravid) [31], dissected into head/thorax and abdomen regions using sterile techniques and stored individuals in Longmire’s lysis buffer solution [32] in preparation for subsequent DNA extraction and blood meal analysis. For female mosquitoes that could not be dissected in the field due to feasibility and time constraints, we stored individual whole mosquitoes in 95% ethanol for subsequent dissections and DNA extraction in the Parker Laboratory at the University of Missouri, Saint Louis, USA. Mosquitoes preserved in ethanol could not be classed according to the Sella scale as the distinct digestive stages of female mosquitoes were often difficult to observe after months of preservation. Nonetheless, the sterile techniques applied to both field and laboratory dissected samples included cleaning hands before each dissection and utilizing a clean slide for every mosquito. Prior to dissecting each individual mosquito, we dipped dissection tools into 10% bleach, rinsed in distilled water, air-dried and applied heat to tools using a Bunsen burner. These techniques were strictly followed to avoid cross-contamination of specimen DNA.

### Blood-meal analysis

Genomic DNA from abdomens of female mosquitoes was extracted using Macherey-Nagel NucleoSpin® Tissue Kit (Macherey-Nagel, Bethleham, USA) according to manufacturer instructions. We used a universal BM primer set developed by Kocher et al. [33]; this primer set amplifies a fragment of 358 bp of the vertebrate cytochrome *b* (*cytb*) gene (forward: 5′-CCC CTC AGA ATG ATA TTT GTC CTC A-3′ and reverse 5′-CCA TCC AAC ATC TCA GCA TGA TGA AA-3′) in assessing sources of mosquito blood meals *via* polymerase chain reaction (PCR). Negative controls were used (all reagents minus template DNA) and showed up as truly negative for all PCR reactions in this study. Positive controls included different taxa representing wildlife DNA samples from Galápagos species. Positive controls consisted of two individuals of marine iguanas (*Amblyrhynchus cristatus*), two species of birds (an introduced bird, the cattle egret *Bubulcus ibis* and an endemic bird, the large ground finch *Geospiza magnirostris*), and finally, two samples from a mammal (*Homo sapiens*). A Takara Taq PCR Kit (Takara Bio USA, Inc., Mountain View, USA) was used for all PCRs according to the manufacturer’s recommendation. The PCR reaction contained 15.875 µl of sterile distilled water, 2.5 µl of 10 × buffer (containing 100 mM Tris-HCl, pH 8.3, 500 mM KCl, 15 mM MgCl_2_), 2 µl of dNTP mix (2.5 mM/l each), 1.5 µl of MgCl_2_ (25 mM), 1 µl of each primer (10 μmol/l), 0.125 µl of Taq (5 U/μl) and 1 µl of extracted DNA template in producing a total volume of 25 µl [34]. Reactions were amplified to the PCR conditions following Hamer et al. [35]. Amplifications were assessed by gel electrophoresis using 1.5% agarose and positive PCR products were purified and sent to Eurofins Genomics LLC (Eurofins Genomics, Louisville, USA) for sequencing.

Sequencing results were subjected to BLAST search in GenBank and each chromatogram was inspected for sequence quality. Applying the rule of parsimony, our criteria involved analyzing sequencing chromatograms showing single peaks at each position as the source of blood meal for arthropod vectors. Mixed blood meals indicated by double or triple peaks on nucleotide chromatograms were removed from the analysis. Furthermore, samples that produced an ambiguous amplicon with no match or with low-quality peaks were re-run with a second reaction using an avian primer set (forward: 5′-GAC TGT GAC AAA ATC CCN TTC CA-3′ and reverse: 5′-GGT CTT CAT CTY HGG YTT ACA AGA C-3′) [34]. This primer set targets a 508-bp fragment size in the *cytb* gene under the reaction conditions described above [34, 35]. If amplicons failed to produce high-quality single peaks, we further subjected samples to a third reaction targeting 772 bp in the mammalian *cytb* gene (primers-forward: 5′-CGA AGC TTG ATA TGA AAA ACC ATC GTT G-3′ and reverse: 5′-TGT AGT TRT CWG GGT CHC CTA-3′) [34]. Reactions also followed the same conditions described above. Samples that produced single peaks in any of the three reactions with a satisfactory match of 98–100% to sequences in GenBank were accepted as the source of origin for mosquito blood meals.

## Results

### Mosquito survey

A total of 1011 mosquitoes were collected in the summer of 2015 over 216 trap nights, consisting of 757 *Ae. taeniorhynchus* and 254 *Cx. quinquefasciatus*. We collected 38 male and 719 female *Ae. taeniorhynchus* (Table 1) and 26 male and 228 female *Cx. quinquefasciatus* (Table 2). Female *Ae. taeniorhynchus* were captured at all but four sites on Santa Cruz. Abundances of female *Ae. taeniorhynchus* were highest in coastal elevations and generally declined with increasing elevation; 40% of female mosquitoes were captured in Puerto Ayora (site 9A and 9B), 14% at site 3A, a site 15 km south of Itabaca Channel, 12% at Itabaca Channel and 14% at Miramar (site 8A and 8B) (Table 1). In contrast, *Cx. quinquefasciatus* female mosquitoes were captured at only 8 sites on Santa Cruz with 60% of captures occurring in Puerto Ayora (site 9A and 9B), 16% at site 8B at Miramar and 6% at Itabaca Channel (site 1A and 1B) and at site 3A (Table 2).

**Table 1:** Summary of wild-caught totals of *Aedes taeniorhynchus* with engorged females and resolved blood meals identified across 18 sites on Isla Santa Cruz, Galápagos

Site	Total male captured	Total female captured	Total blood-fed mosquitoes	Total resolved blood meals
1A	0	23	0	0
1B	2	71	33	31
2A	0	0	0	0
2B	0	1	0	0
3A	5	105	44	41
3B	0	0	0	0
4A	0	0	0	0
4B	0	0	0	0
5A	2	18	4	4
5B	0	5	0	0
6A	0	21	18	18
6B	1	47	23	22
7A	0	4	0	0
7B	0	4	0	0
8A	2	2	0	0
8B	1	109	21	20
9A	3	202	55	52
9B	22	107	47	44
Total	38	719	245	232

**Table 2:** Summary of wild-caught totals of *Culex quinquefasciatus* with engorged females and resolved blood meals identified across 18 sites on Isla Santa Cruz, Galápagos

Site	Total male captured	Total female captured	Total blood-fed mosquitoes	Total resolved blood meals
1A	0	3	3	2
1B	4	11	0	0
2A	0	0	0	0
2B	0	0	0	0
3A	5	14	4	4
3B	0	0	0	0
4A	0	0	0	0
4B	0	0	0	0
5A	0	1	0	0
5B	0	0	0	0
6A	0	0	0	0
6B	0	5	2	2
7A	0	0	0	0
7B	0	0	0	0
8A	0	0	0	0
8B	0	41	2	1
9A	12	52	15	14
9B	5	101	49	46
Total	26	228	75	69

### Blood-meal analysis

**Fig. 2:**
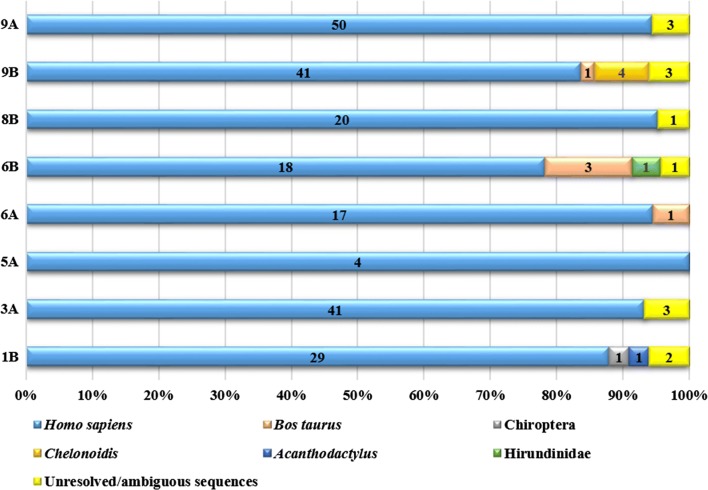
Host and site feeding range of *Aedes taeniorhynchus*. Numbers indicated in colored bars represent counts of resolved blood meals and numbers in yellow bars represent counts of unresolved/ambiguous sequences. *Homo sapiens*, *Bos taurus* and Chiroptera represent mammalian families. *Chelonoidis* and *Acanthodactylus* represent reptilian families and Hirundinidae represents an avian family. Y-axis represents trapping sites across Santa Cruz and X-axis represents proportion of blood meals from total numbers of mosquitoes captured/site

Out of 719 female *Ae. taeniorhynchus* mosquitoes, molecular screening identified 245 females as positive for taking a blood meal from a vertebrate host. Of these, 232 *Ae. taeniorhynchus* blood meals were resolved with sequencing chromatograms showing single high-quality peaks at each position. Thirteen blood meal sources remained unresolved and either failed to amplify even after multiple PCR attempts (Table 1). We identified 95% (220 mosquitoes) of blood meal sources as originating from humans (*Homo sapiens*), 2% (5 mosquitoes) from cattle (*Bos taurus*) and 1.7% (4 mosquitoes) from Galápagos tortoises (*Chelonoidis* spp.) (Fig. 2). A blood meal from one mosquito captured at site 6B in Santa Rosa (381 masl) contained DNA from a bird belonging to the family Hirundinidae and a 100% match to *Tachycineta bicolor*. Another *Ae. taeniorhynchus* mosquito captured at site 1B on Itabaca Channel was identified as having taken a blood meal from a reptile (Class Reptilia, Order Squamata). A blood meal from one *Ae. taeniorhynchus* mosquito captured at site 1B on Itabaca Channel was identified as having fed from a mammal in the order Chiroptera (bats) (Fig. 2). Humans were detected as a source of blood meal in mosquitoes captured both in southern and northern Santa Cruz and at low and high elevations. The largest number of mammalian blood meals, including 91 mosquitoes detected with human blood meals, was recorded at Puerto Ayora (site 9A and 9B), populated with nearly 12,000 human inhabitants. Mosquitoes with humans as a source of blood meal were captured at elevations of ~ 300 masl and at the highest elevation site in Los Gemelos (site 5A, 618 masl). Cattle (*Bos taurus*) as a source of blood meals were identified in 4 mosquitoes captured in Santa Rosa (site 6A and 6B) and in one mosquito captured at site 9B in Puerto Ayora. All mosquitoes identified with blood meals from Galápagos tortoises (*Chelonoidis* spp.) were captured at site 9B in Puerto Ayora (Fig. 2); there is a captive breeding program for tortoises at the Galápagos National Park headquarters located just outside of Puerto Ayora.

**Fig. 3:**
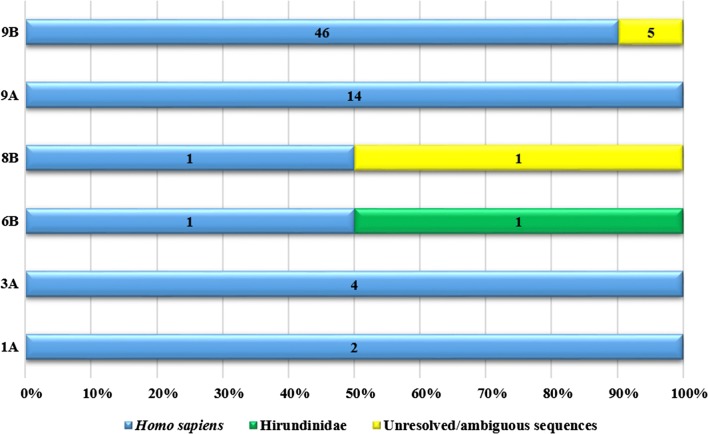
Host and site feeding range of *Culex quinquefasciatus*. Numbers indicated in colored bars represent counts of resolved blood meals and numbers in yellow bars represent counts of unresolved/ambiguous sequences. Y-axis represents trapping sites across Santa Cruz and X-axis represents proportion of blood meals from total numbers of mosquitoes captured/site

For a total of 228 female *Cx. quinquefasciatus* mosquitoes captured, molecular screening identified 75 mosquitoes with blood meals. Of these, 69 mosquitoes had blood meals that were resolved with chromatograms showing single high-quality peaks, indicating a single source of blood meal from a vertebrate species (Table 2). A total of 68 out of 69 of these blood meals were identified as human with 87% (*n* = 60) of blood-fed mosquitoes captured in Puerto Ayora (site 9A and 9B) alone (Fig. 3). We identified a single human-fed *Culex* mosquito at site 8B in Miramar, located 5 km north of Puerto Ayora and at site 6B, located at Santa Rosa. Mosquitoes identified with human blood meals were also captured at northern sites 3A and at the most northern site of Itabaca Channel (site 1A). One mosquito captured at site 6B was identified as positive for having a blood meal from a bird belonging to the family Hirundinidae with a 100% match to *Tachycineta bicolor*.

## Discussion

Our analysis of the blood-feeding behavior of mosquitoes gives insight into their roles as disease-carrying vectors on an inhabited island in Galápagos. We found that both *Ae. taeniorhynchus* and *Cx. quinquefasciatus* are widespread and that sites with the highest abundances of blood-fed female mosquitoes are those that record high mosquito abundances in general. The number of blood meals from *Ae. taeniorhynchus* was three times that of *Cx. quinquefasciatus* and this corresponded to the sample size of female mosquitoes of each species collected in the summer of 2015. Since we sampled in the dry season of 2015, it is not surprising that we generally captured low numbers of *Cx. quinquefasciatus*, a species whose females require freshwater to oviposit eggs. Most *Cx. quinquefasciatus* mosquitoes were captured in areas of human settlements and this is not surprising given it is a freshwater obligate [36]. *Culex* mosquitoes have often been associated with human populations who provide conducive environments for mosquito larval development *via* stagnant rainwater in old tires, ditches, drains, tanks, or containers [37]. On the other hand, since *Ae. taeniorhynchus* females oviposit in brackish water, their relatively high abundances in our study could be attributed to the availability of mangrove habitats as well as ideal environmental conditions conducive for mosquito breeding [38]. In general, the abundances and distributional patterns of both mosquito species follow similar patterns to previous studies in Galápagos and can influence disease transmission dynamics amongst native avifauna [13, 15, 38, 39].

*Aedes taeniorhynchus* has been shown to feed primarily on mammals and reptiles in Galápagos [12]. Our study supports this finding with 99% of blood meals identified from mammalian and reptilian hosts and included humans, bats, cattle, land tortoises and lava lizards. The only non-reptilian/non-mammalian blood meal was identified as *Tachycineta bicolor* (tree swallow) which could be a vagrant in Galápagos. The mosquito blood meal could also be from other birds in the family Hirundinidae such as the endemic Galápagos martin (*Progne modesta*), which is found in the highlands of the central and southern islands of the archipelago or the purple martin (*Progne subis*), an infrequent visitor.

Mammalian blood meals were highest in our study with 96% of engorged *Aedes* females identified as having fed from mammals. Bataille et al. [12] also found that *Ae. taeniorhynchus* mosquitoes in Galápagos prefer mammals and reptiles over birds. Unfortunately, results from our research cannot support *Ae. taeniorhynchus* as having a preference due to the study’s limitations in lacking data on host abundance and mosquito preference. However, since mammal blood meals were found across the island of Santa Cruz, this can indicate that *Ae. taeniorhynchus* feeding behavior on mammals is widespread. In areas with human settlements such as in Puerto Ayora, Miramar and Santa Rosa, numbers of engorged mosquitoes were highest, indicating humans as an important source of blood meals for mosquitoes. We also found a high proportion of human blood meals in mosquitoes captured at Itabaca Channel, which is the point of entrance for tourists or visitors to Santa Cruz and Galápagos. Both *Ae. taeniorhynchus* and *Cx. quinquefasciatus* feed primarily at night and our night-time trapping protocol allowed us to sample when humans were less active and mosquito blood-feeding behaviors were highest. The majority of blood meals in our study originated from humans, whose abundance we did not assess at our capture sites; therefore, we did not include any analysis of preference. However, we do recommend that future sampling of mosquitoes and vertebrate hosts be conducted during diurnal periods as well to better quantify host abundance and determine mosquito preference by use of the foraging ratio analysis [40], which estimates the significance of host blood meal preference as a function of the relative abundance of different host species. In addition, we recommend a systematic sampling of mosquitoes and hosts in uninhabited islands to gain a better understanding of mosquito feeding preferences in and across the Galápagos archipelago.

We also captured blood-fed *Ae. taeniorhynchus* and *Cx. quinquefasciatus* at uninhabited sites, Los Gemelos (site 5A) and site 3A, suggesting dispersal or movement of mosquitoes throughout the island of Santa Cruz. Mosquitoes have been known to disperse between and within islands in Galápagos through human-aided transportation such as airplanes and boats [41] and the availability of a well-developed road network in Santa Cruz could further facilitate the movement of mosquitoes. *Aedes taeniorhynchus* is known to disperse up to 40 km [42] while *Cx. quinquefasciatus* can travel up to 3 km [43–45] and their long-range dispersal could further broaden the geographical range of wildlife pathogens.

Adult female *Ae. taeniorhynchus* feed primarily at night and are hematophagous (or blood-feeders), while males may nectar-feed [46]. Female mosquitoes utilize blood from vertebrate species to develop their eggs; however, this species is partially autogenous, meaning that it can oviposit an initial batch of eggs without a blood meal [47]. Even though a blood meal is not a pre-requisite for egg production in *Ae. taeniorhynchus*, autogenous females readily consume a blood meal during the first and second day following emergence and blood-feeding can significantly increase egg production [48]. Abundant vertebrate species such as mammals and reptiles in Galápagos provide a readily available foraging resource for partially autogenous *Ae. taeniorhynchus* females in producing a large initial egg batch, which leads to high mosquito abundances for this species. Hence, if the relatively large non-avian host population contributes to overall egg production and mosquito abundances, disease transmission may generally be amplified by mosquitoes, particularly if they are competent arthropod vectors. This amongst many factors such as infection rate, availability of sites for the development of mosquito larvae and abiotic factors such as rainfall and temperature would result in a greater risk of disease transmission of parasites such as avian malaria to native birds, compared to what would be expected in areas of low mammalian and reptilian host abundances.

Examination of blood-fed mosquitoes in our study showed an almost exclusively mammalian diet of *Cx. quinquefasciatus* on Santa Cruz. With the exception of one blood meal from a bird belonging to the family Hirundinidae, all analyzed blood meals were identified as human. Our study may support research that indicates that *Cx. quinquefasciatus* is an inherent opportunistic feeder [49] and a generalist feeder, meaning that it feeds indiscriminately on both birds and mammals [50]. However, our results need to be interpreted with caution given the absence of a foraging ratio analysis. Our findings may also indicate humans as one of the most abundant host species that is locally available, but this does not necessarily mean that it is the preferred host. For instance, blood meal screening from *Cx. quinquefasciatus* captured in Kenya revealed only 3–9.8% of human blood meals; the majority of blood meals originated from other mammals such as cattle, goats and donkeys [51]. In Tanzania, experimentation with an equal availability of three vertebrate species found *Cx. quinquefasciatus* behavior as highly anthropophilic [52]. In other sites, *Cx. quinquefasciatus* has also been shown to generally prefer feeding on birds [50] and occasionally on reptiles, amphibians, and mammals [53, 54]. In northeastern Mexico, foraging ratios of *Cx. quinquefasciatus* were highest for chickens compared to humans, horses and pigs and this was attributed to chickens being highly abundant in the area of study [55]. Sites included in our trapping scheme which fall in agricultural zones include Bellavista and Santa Rosa, both located on southern slopes of Isla Santa Cruz. During trapping nights at both locations, our mosquito traps were placed closer to human settlements than to agricultural sites and therefore could have resulted in the greater detection of human blood meals than from farm animals such as chickens, pigs and cows at nearby farms. Nevertheless, the high plasticity in feeding behavior in *Cx. quinquefasciatus* could indicate that it may be an opportunistic feeder as referenced in many studies above and that its feeding behavior varies with locally available and abundant species. However, without a proper estimation of host abundances and feeding preferences of mosquitoes in Galápagos, caution must be applied, as the findings from other mosquito blood meal studies might not be transferable to mosquitoes in our study area. In addition, realizing that our research lacks an abundance estimate of different fauna to be utilized in a foraging ratio analysis, we cannot say with confidence that any particular species is highly abundant or is preferred as a blood meal source by mosquitoes in Santa Cruz.

Nevertheless, even though mammals made significant contributions to the blood meals of *Cx. quinquefasciatus* and *Ae. taeniorhynchus*, both mosquitoes also fed on other non-mammalian vertebrate species. The plasticity of mosquitoes in Galápagos to feed on different vertebrate blood meal sources could give us clues to the transmission of wildlife pathogens among hosts. For instance, if mosquitoes feed broadly on a range of non-avian host species, the chance of detecting avian parasites is small. The avian malaria parasite (*Plasmodium* spp.) has a very low infection rate in Galápagos and may be difficult to detect, particularly if competent vectors such as *Cx. quinquefasciatus* are not abundant and are feeding mostly on non-avian hosts such as mammals and reptiles. In fact, *Culex* mosquitoes have been shown to modify their feeding preferences based on host availability and abundance and provide a bridge in the transmission of West Nile virus (WNV) from birds to humans [34, 35]. A detailed study integrating feeding behavior of mosquitoes and composition of host species showed that American robins, which are competent WNV hosts, were preferentially fed on by the mosquito species *Culex tarsalis*. However, during periods of robin dispersal and migration, *Cx. tarsalis* shifted its feeding preferences from birds to humans. This greatly amplified the number of human infections, particularly when mosquito infection prevalence was high from feeding on infected robins [56]. *Culex quinquefasciatus* has the capacity to transmit avian malaria [1] but the low malarial infection rate and generalist feeding behavior of *Culex* could be minimizing the chances of detecting *Plasmodium* in Galápagos mosquito sampling. Additional studies investigating the feeding preferences of mosquitoes on islands without human populations along with experimental infection of hosts and arthropod vectors are recommended to resolve this question.

## Conclusions

Our study assessed the feeding patterns of two common mosquito species, *Ae. taeniorhynchus* and *Cx. quinquefasciatus* in the inhabited island of Santa Cruz, Galápagos. Our results indicated a high proportion of mammalian blood meals in both species, which may reflect locally available and abundant hosts in Santa Cruz. However, surveys documenting the relative abundances of hosts as potential sources of mosquito blood meals will need to accompany mosquito trapping studies to further validate this. Determining the host feeding range of mosquitoes and their feeding preferences is critical to understanding the disease dynamics of wildlife pathogens such as avian malaria. This knowledge is important in contributing towards managing pathogens that threaten the conservation of endemic wildlife, particularly avifauna in isolated islands such as Galápagos.

## Data Availability

Data supporting the conclusions of this article are included within the article.

## Abbreviations

BM: blood meal
CA: California
CDC: Centers for Disease Control, USA
CDF: Charles Darwin Foundation
dNTP: deoxyribonucleotide triphosphate
FRC: Field Research for Conservation Program, Saint Louis Zoo
GNP: Galápagos National Park
IUCN: International Union for Conservation of Nature
KCl: potassium chloride
masl: meters above sea level
MgCl_2_: magnesium chloride
PCR: polymerase chain reaction
Tris-HCl: Tris (hydroxymethyl) aminomethane hydrochloride
USDA APHIS: United States Department of Agriculture-Animal and Plant Inspection Service
UMSL: University of Missouri Saint Louis
WNV: West Nile virus
